# Reversible Sol–Gel Transition in Thermoresponsive Collagen Hydrogels for Cryogen-Free Cell Logistics

**DOI:** 10.3390/gels12060488

**Published:** 2026-06-02

**Authors:** Junjie Wang, Yi Ju, Yang Lei, Jieyu Zhang, Yunbing Wang

**Affiliations:** 1 National Engineering Research Center for Biomaterials, College of Biomedical Engineering, Sichuan University, No. 29 Wangjiang Road, Chengdu 610065, China; 2 Institute of Biomedical Innovation, School of Basic Medical Sciences, Jiangxi Medical College, Nanchang University, No. 1299, Xuefu Road, Honggutan District, Nanchang 330031, China

**Keywords:** live cell transport, cryogen-free transportation, thermosensitive hydrogel, collagen, 3D cell culture, cell recovery

## Abstract

Cell culture is foundational to biomedical advancements, yet its widespread clinical and practical distribution is severely constrained by the high infrastructural costs of cryogenic logistics and the physical stressors of liquid-phase transit. Herein, we propose a proof-of-concept cryogen-free cell transportation strategy leveraging a rapid reversible thermoresponsive collagen (RRTC) hydrogel regulated by simulated body fluid (SBF). Operating via temperature-driven physical network assembly and disassembly rather than chemical crosslinking or chemical modifications, the RRTC system undergoes a rapid sol-to-gel transition within 60 s at 37 °C for efficient cell encapsulation, and completely reverses to a free-flowing sol state within 60 s at 4 °C to facilitate enzyme-free, non-destructive cell retrieval. Using L929 fibroblasts as a standardized benchmarking cell model, the biophysical protection of the matrix was systematically evaluated under both static simulated transit (48 h and 120 h) and real-world trans-city courier transportation (an approximate 50 h round trip via SF Express) within a passively temperature-shield configuration. The SBF-regulated 3D physical confinement successfully shielded cells from manual handling, multi-axis shipping vibrations, and environmental thermal fluctuations. Post-transport evaluations demonstrated that the encapsulated cells maintained a high viability above 90% and a stable recovery yield of approximately 78%, while exhibiting robust subsequent 2D re-adhesion and sustained re-culture capacity. This thermoresponsive matrix provides a potential matrix for short-term cryogen-free cell transportation and post-transport recovery, while further studies using additional cell types, longer transportation periods, and functional assays are required to evaluate its broader applicability.

## 1. Introduction

Cell culture serves as the fundamental cornerstone for all biomedical research and biopharmaceutical advancements, including the rapidly expanding cell-based therapies [1]. However, a critical bottleneck restricting the widespread clinical translation and practical accessibility of these live-cell technologies is that conventional distribution relies heavily on cryogenic logistics. While effective for long-term preservation, this cold chain is plagued by high financial costs, complex handling protocols, and strict reliance on specialized ultra-low-temperature infrastructures, which imposes an inequitable economic and logistical burden on remote areas and developing regions (i.e., in Africa). More importantly, the requisite freezing–thawing cycles expose cells to severe osmotic stress and intracellular ice crystal formation, frequently resulting in impaired biological functionality and significant cell loss [2]. Furthermore, beyond these low-temperature constraints, cells remain highly sensitive to environmental and mechanical stresses encountered during transit, where uncontrolled shipping vibrations and physical shocks further jeopardize cellular homeostasis.

To address these cryogenic hurdles, non-cryogenic transport under controlled or buffered conditions has been used [3,4,5,6,7]. Nevertheless, merely shifting away from freezing temperatures does not safeguard cells against transit-induced mechanical disruptions. In conventional two-dimensional (2D) liquid suspension conditions, cells suffer from a lack of biomechanical support and extracellular matrix (ECM) cues. Without a protective matrix to anchor the cells against shipping vibrations and physical shocks, cells in liquid media undergo cell rounding, detachment, and anoikis (detachment-induced apoptosis), which severely restricts the available transportation window [8,9]. Successfully mitigating these physical and biological stressors necessitates an engineered microenvironment that mimics the native ECM to preserve cellular homeostasis during transportation. In this manuscript, cryogen-free refers to transportation conducted without dry ice, liquid nitrogen, or active cryogenic refrigeration, although passive temperature buffering may be used to avoid overheating during transport.

To provide this essential biomimetic protection, three-dimensional (3D) hydrogel biomatrices have emerged for non-cryogenic cell logistics [10]. These viscoelastic networks provide essential physical encapsulation, mitigate fluid shear stress during transit, and replicate physiological cell–matrix interactions to maintain cellular homeostasis [11,12]. While several pioneering studies have explored non-cryogenic cell carriers using alginate [13], specialized phospholipid polymers [14], gelatin [15], and self-assembling peptides [16], as well as stimuli-responsive hydrogels based on collagen [17], gelatin [18], Gelatin methacryloyl (GelMA) [19], or collagen–polyethylene glycol (PEG) [20], their widespread translation and commercial viability remain severely hindered by a fundamental paradox between structural stabilization and cell harvesting. Many of these systems heavily rely on covalent chemical modification, photo-crosslinking, radical polymerization, or exogenous ionic crosslinking, to achieve the network stability required to withstand transit handling. Consequently, cell retrieval from these carriers inevitably requires either complex chemical triggers or harsh enzymatic digestion for cell recovery [21]. These post-transport processing steps remain a major barrier to practical application because they may prolong handling time, induce secondary cell damage, and affect post-recovery cell phenotype [22,23]. Although specific thermo-responsive collagen composite matrices have been reported for 3D biomedical applications such as organoid culture [17], these established platforms are primarily optimized as static structural scaffolding for long-term incubation, where their self-assembly and dissociation kinetics are not tailored for rapid matrix regression. Consequently, they remain fundamentally distinct from an integrated short-term cell logistics platform that inherently couples sub-minute in situ encapsulation, passively temperature-buffered transportation, and completely enzyme-free cell retrieval.

Collagen, as a major structural component of the native ECM, offers excellent biocompatibility and intrinsic bioactivity. The unique triple-helical architecture of collagen actively supports and regulates cell adhesion, migration, spreading, and proliferation [24,25], making it an ideal candidate for biomimetic engineering. In the present study, we investigated a type I collagen-based rapid reversible thermoresponsive collagen (RRTC) hydrogel regulated by simulated body fluid (SBF). In this system, SBF provides a physiologically relevant electrolyte environment that regulates the hydration shells and electrostatic screening around the collagen triple-helices, thereby guiding the assembly of the physical network. Previous investigations indicated that the molecular weight and native triple-helical conformation of collagen were successfully retained in this formulation, suggesting that the reversible phase transition is governed by a temperature-driven physical network assembly–disassembly process rather than irreversible chemical crosslinking or macromolecular degradation. At 4 °C, low molecular thermal mobility combined with a stabilized hydration layer maintains a free-flowing sol state. Upon warming to 37 °C, increased chain mobility and enhanced hydrophobic interactions overcome the hydration barrier, driving rapid, physically crosslinked network formation within 60 s for efficient in situ cell encapsulation. Conversely, post-transport treatment with a pre-cooled medium at 4 °C destabilizes these weak physical junctions to facilitate prompt physical network dissociation and gentle, non-destructive cell recovery.

**Scheme 1:**
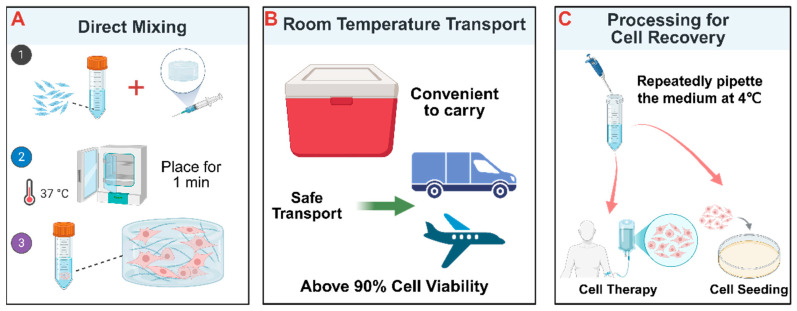
Schematic illustration of the cryogen-free, passively temperature-buffered cell transportation strategy based on a thermoresponsive collagen hydrogel. (**A**) L929 fibroblasts were mixed into the precursor sol and rapidly encapsulated in situ via a temperature-triggered sol–gel transition at 37 °C. (**B**) The resulting 3D hydrogel matrix provided ECM-like structural support during the tested transportation conditions. (**C**) The intrinsically reversible thermal responsiveness of the hydrogel allows for enzyme-free cell recovery after post-transport treatment with pre-cooled medium at 4 °C. Under the designated conditions (encompassing 48 h and 120 h static simulated transits as well as an approximate 50 h real-world trans-city courier round trip), this integrated platform maintained a high post-transport cell viability (>90%) for the model L929 fibroblasts.

Herein, we propose a proof-of-concept cryogen-free cell transportation strategy leveraging this reversible thermoresponsive collagen network. By encapsulating cells within this 3D collagen-based matrix, the system shields them from the detrimental stresses of liquid-phase transit while providing ECM-like structural support. We systematically evaluated the biophysical properties of the matrix and investigated the post-transit viability, recovery efficiency, and sustained re-culture capacity of the encapsulated cells under static simulated and real-world courier conditions. The primary novelty of this work lies in its application-level and workflow-integration innovations, moving away from conventional cryogenic storage to address practical bottlenecks in short-term cell logistics. Specifically, this integrated platform bypasses the heavy infrastructural costs and ice sublimation constraints of traditional cold chains by establishing a reliable ambient or room-temperature logistics workflow under passive temperature buffering. Furthermore, by verifying this delivery loop via a standard commercial courier network (an approximate 50 h trans-city round trip via SF Express), we demonstrate that the 3D physical confinement successfully protects cells from handling, multi-axis vibrations, and environmental fluctuations, maintaining high viability and robust re-culture ability while providing an enzyme-free loop. Using L929 fibroblasts as a standardized cell model, we evaluated post-transport viability, recovery efficiency, and re-adhesion after static simulated transportation and real-world courier transportation (Scheme 1).

## 2. Results and Discussion

### 2.1. Sol–Gel Phase Transition and Thermoresponsive Rheology of RRTC

**Figure 1:**
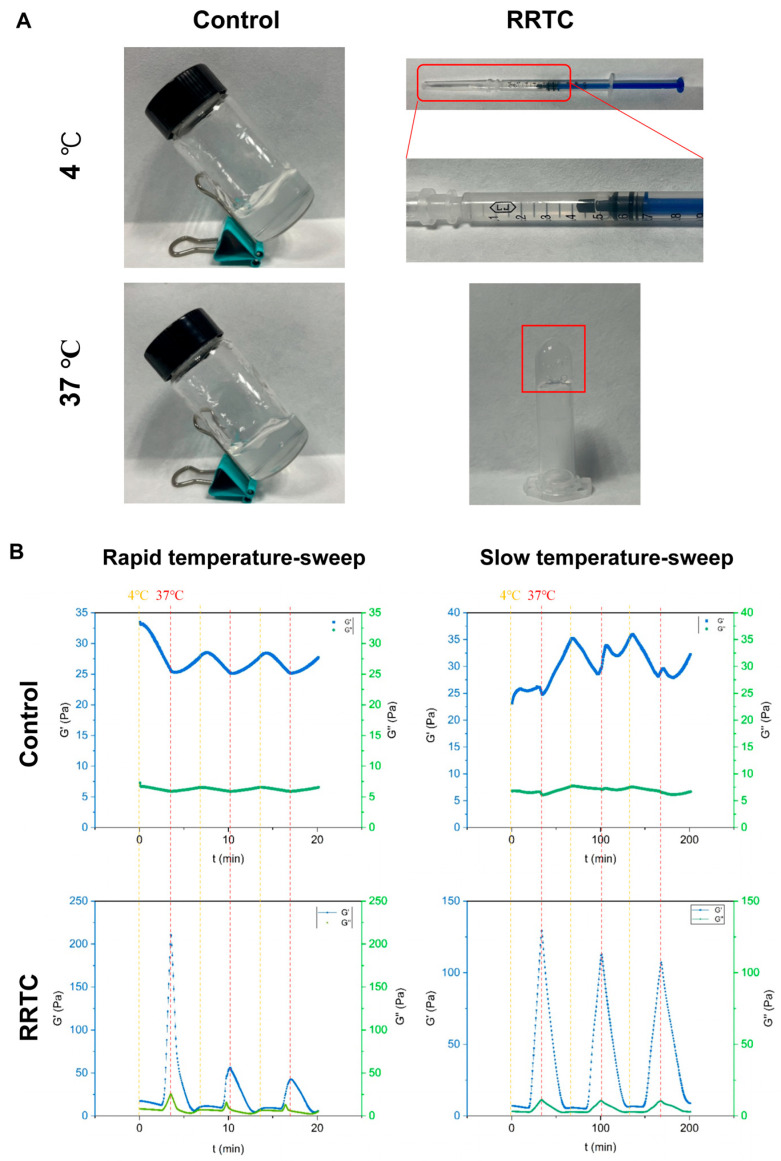
Characterization of the thermoresponsive properties of the RRTC hydrogel. (**A**) Macroscopic reversible phase-transition behavior of the type I collagen control hydrogel and the RRTC hydrogel at 4 °C and 37 °C. The red square indicates the RRTC hydrogel. (**B**) Thermoresponsive rheological properties of the type I collagen control hydrogel and the RRTC hydrogel during repeated heating–cooling cycles.

To address the biophysical and logistical limitations of conventional cell distribution outlined above, we employed a rapid, reversible, thermoresponsive collagen (RRTC) hydrogel regulated by simulated body fluid. The injectable RRTC precursor solution (Video S1) exhibited a temperature-dependent phase-transition behavior. Upon heating from 4 °C to 37 °C, the precursor underwent a rapid complete sol-to-gel transition in less than 60 s, forming a gel-like RRTC hydrogel. This macroscopic phase transition was reversible. When the temperature was decreased from 37 °C to 4 °C, the preformed RRTC hydrogel reverted into a free-flowing sol state in less than 60 s. By comparison, the bovine type I collagen control failed to exhibit any discernible reversible sol–gel conversion under identical thermal cycling, indicating the distinct thermoresponsive behavior of the RRTC system (Figure 1A).

To elucidate the underlying viscoelastic dynamics of this thermoresponsive behavior, rheological evaluations were performed. During the heating sweep, the RRTC hydrogel demonstrated a marked increase in storage modulus (G’) while the loss modulus (G″) exhibited a more gradual increment, suggesting that the system progressively shifted from a viscosity-dominated state to an elasticity-dominated state, reflecting physical network formation (Figure 1B). Upon cooling, the decrease in G’ indicated partial disassembly of the gel network and recovery toward the sol state. Notably, under slow temperature-sweep cycles, the storage modulus of the RRTC matrix exhibited some attenuation after multiple temperature cycles but remained relatively high overall, indicating apparent cyclic responsiveness with partial attenuation. Under rapid temperature-sweep cycles, the modulus peaks of the RRTC matrix gradually decreased in subsequent cycles, suggesting that rapid heating and cooling may partially hinder the complete recovery of the gel network. This attenuation suggests that the reversibility observed here mainly reflects macroscopic phase behavior rather than complete molecular-level reversibility. In contrast, the bovine type I collagen exhibited negligible thermal responsiveness, maintaining a plateaued G′ of merely 25–40 Pa throughout the thermal cycles. These rheological results indicate that the RRTC matrix exhibits a temperature-dependent viscoelastic profile, a prerequisite for rapid cell encapsulation and on-demand recovery.

To provide insight into the underlying molecular and structural factors governing this distinct thermoresponsive behavior, the macroscopic phase transitions can be interpreted in terms of the physicochemical interactions between the collagen architecture and the SBF microenvironment. According to the related literature, the type I collagen within this SBF-regulated formulation successfully retains its native molecular weight and triple-helical conformation [26,27]. Building upon these established structural parameters, the rapid reversible sol–gel transition observed in our rheological and macro-scale analyses is governed by a temperature-driven physical assembly disassembly process rather than chemical crosslinking or macromolecular degradation. SBF introduces a physiological ionic strength and a specific electrolyte composition. This biomimetic ionic microenvironment provides critical electrostatic screening that regulates the hydration shells surrounding the collagen triple-helices. At 4 °C, the low molecular thermal mobility and stabilized hydration layers maintain the system in a free-flowing sol state. Upon heating to 37 °C, increased chain mobility and enhanced hydrophobic interactions overcome this hydration barrier, driving the rapid formation of a physically crosslinked network within 60 s. Conversely, post-transport treatment with a pre-cooled medium at 4 °C destabilizes these weak physical junctions and restores the hydration layer, facilitating complete physical network dissociation for enzyme-free cell retrieval. While direct in situ, time-resolved molecular tracking during the exact phase transition window remains an objective for future specialized investigations, this literature-supported physical model offers a cautious and reasonable explanation for the system’s rapid interfacial reversibility.

The precursor concentration of the RRTC hydrogel plays a fundamental role in dictating its thermoresponsive sol–gel transition kinetics and the resulting network density. From a macromolecular perspective, collagen concentration governs the spatial density of triple-helical templates within the SBF microenvironment, thereby directly altering the frequency of intermolecular physical crosslinking. Higher precursor concentrations increase the availability of physical junction zones, thereby accelerating the temperature-triggered self-assembly kinetics and elevating the storage modulus (G’). In this study, a final collagen concentration of 1.4 wt% was identified as the optimized formulation, balancing the trade-off between mechanical shielding during transit and resistance to post-transport handling. This specialized density enables rapid cell entrapment upon warming, while maintaining a low-viscosity threshold under pre-cooled conditions to facilitate gentle cell retrieval.

### 2.2. 3D Microenvironment of the Cell-Laden RRTC Hydrogel

**Figure 2:**
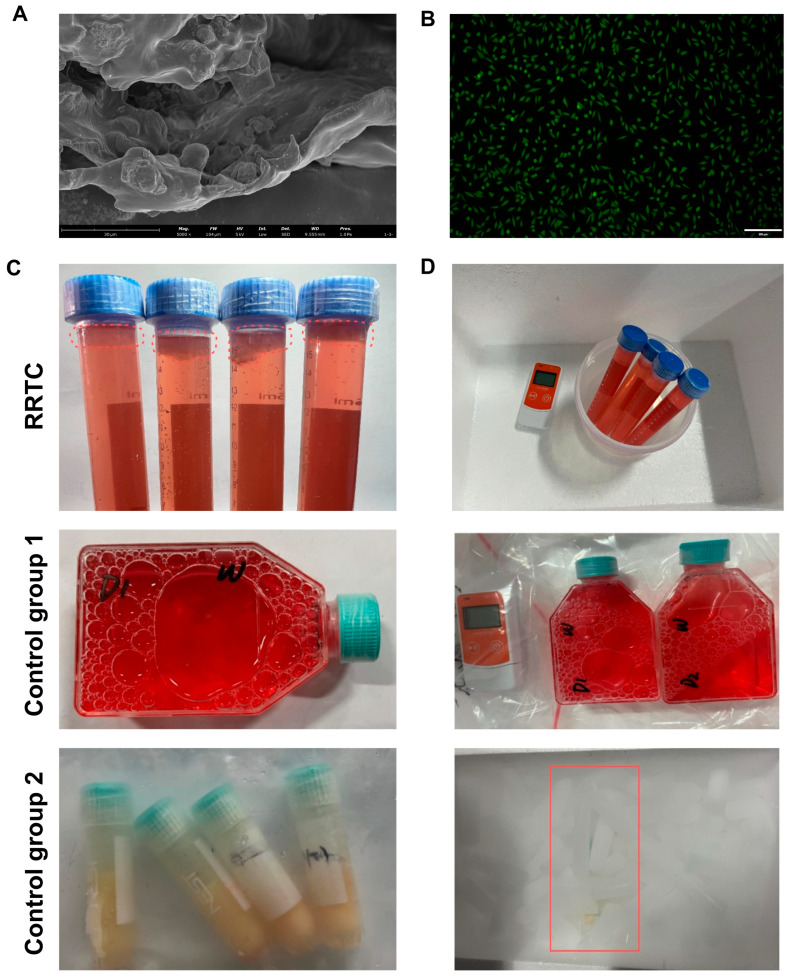
Characterization of the static simulated transportation experiment. (**A**) SEM image of the freeze-dried cross-section of the cell-laden RRTC hydrogel. (**B**) Live/dead staining of cells before simulated transit. (**C**) Photographs of the loading containers used for static simulated transportation in different groups. The area circled with a red dashed line is the RRTC/cell mixture. (**D**) Photographs of the storage conditions for different groups during static simulated transportation. The red square indicates the precise location where the frozen cells were submerged in dry ice.

Beyond physical tunability, evaluating the freeze-dried cross-sectional morphology of the cell-laden matrix is vital for subsequent transportation efficacy. Scanning electron microscopy (SEM) images of freeze-dried cross-sections showed that encapsulated cells were retained within the RRTC hydrogel, with visible multicellular aggregates (Figure 2A). This observation suggests that the RRTC hydrogel can provide a 3D matrix for cell confinement. Because SEM preparation involves freezing, fracture, dehydration, and drying, the observed morphology may include preparation-related artifacts and should not be interpreted as the fully native hydrated hydrogel architecture. The presence of cell aggregates at high cell density may affect oxygen and nutrient diffusion, as well as subsequent cell recovery efficiency, both of which require further optimization.

### 2.3. Cryogen-Free Cell Transportation Under Static and Real-World Courier Conditions

To isolate the hydrogel’s physical shielding performance from cell-type-specific biological variations across complex logistics environments, L929 fibroblasts were utilized as a standardized benchmarking model. (ISO 10993-5 [28]) Mastering this workflow in a single, highly reproducible cell line is a prerequisite for validating the platform on more sensitive therapeutic cell types. To evaluate the feasibility of this platform, a static transportation model was first established. Prior to simulated transit, L929 fibroblasts exhibited intact morphology, good spreading, and favorable baseline viability (Figure 2B). Notably, the RRTC hydrogel encapsulating L929 fibroblasts (2 × 10^7^ cells/mL) maintained its structural integrity without dissolving, even when immersed in excess culture medium, indicating structural stability in fluidic environments (Figure 2C and Video S2).

**Figure 3:**
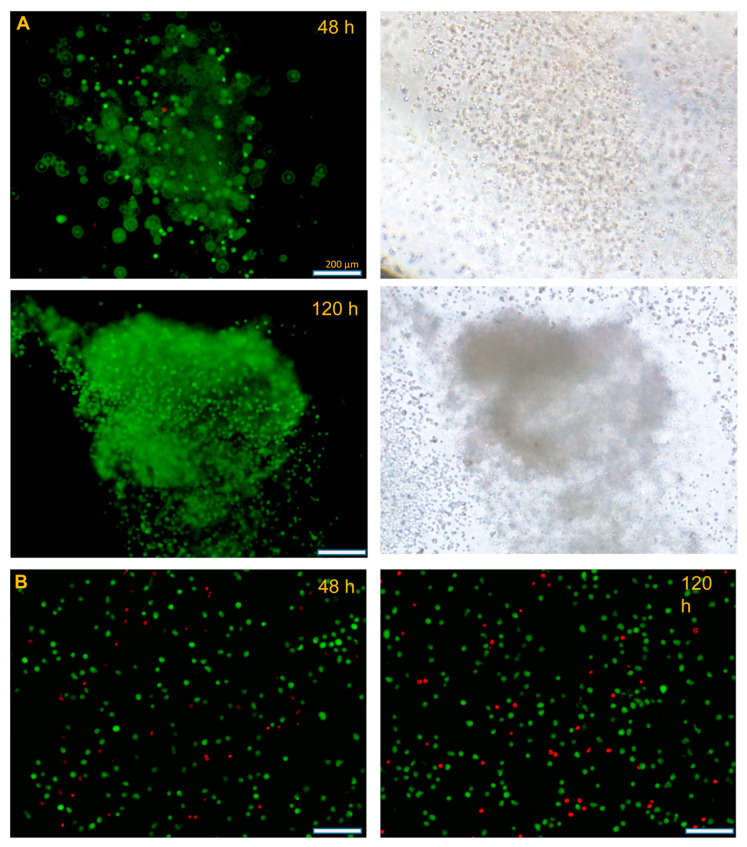
Live/dead staining results of cells in different groups after static simulated transportation. (**A**) Live/dead staining and corresponding bright-field images of cells encapsulated with the RRTC hydrogel after 48 h and 120 h of static simulated transportation. (**B**) Live/dead staining images of the 2D liquid-filled control group after 48 h and 120 h of static simulated transportation. Green and red fluorescence denote viable and dead cells, respectively.

During static simulated transportation spanning 48 h and 120 h (Figure 2C,D), the RRTC matrix demonstrated an ability to maintain cell viability. Live/dead fluorescence assays revealed that cell viability in the RRTC group remained high (Figure 3A). Consistently, bright-field microscopy confirmed that the hydrogel effectively restricted passive cellular dispersion, sustaining stable, multi-layered cellular aggregations within the 3D network.

In contrast, cells in the 2D liquid-filled flasks (Control Group 1) showed increased cell death and reduced viability due to the absence of a supportive niche (Figure 3B and Figure S1). When compared with the conventional cryopreservation-based transport mode (Control Group 2: pre-frozen at −80 °C and placed with 5 kg of dry ice), the RRTC platform showed a potential practical advantage under the tested conditions. Specifically, by 48 h of transit, the dry ice in Control Group 2 had already been largely depleted, and by 120 h it had completely sublimated (Figure S2), indicating the limited capacity of this transport mode to maintain a stable low-temperature environment during prolonged transit. Consistently, live/dead fluorescence staining after thawing showed that dead cells predominated in this group (Figure S3), suggesting that both freeze–thaw injury and the progressive loss of cold-chain protection during transportation reduced cell viability. Because the cryogenic condition was not maintained at later time points, Control Group 2 was interpreted as a practical reference for dry-ice transport failure under the tested packaging conditions rather than as a fully equivalent comparator for all endpoints. Together, these findings suggest the potential advantages of the RRTC-based passively temperature-buffered transport strategy under the tested conditions.

**Figure 4:**
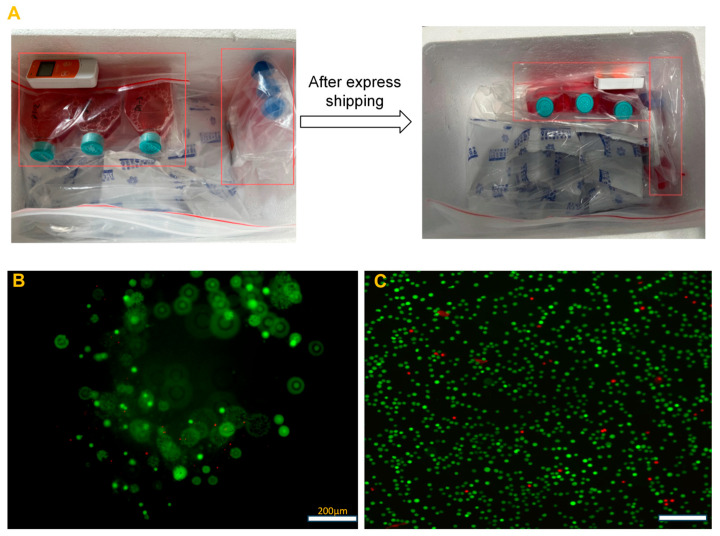
Characterization of real-world courier transportation experiment. (**A**) Photographs of the packaging setup and storage microenvironment for the samples before and after actual courier transportation. Ice packs were used as passive temperature buffers to prevent overheating rather than as a conventional 2–8 °C cold-chain system. (**B**) Live/dead staining images of encapsulated cells within the RRTC hydrogel after transportation. (**C**) Live/dead staining images of cells in the 2D liquid-filled control group after transportation. Green and red fluorescence denote viable and dead cells respectively.

To bridge the gap between static simulations and practical transportation, the constructs were subjected to dynamic courier logistics (a ~50 h round trip between Chengdu and Chongqing via SF Express) (Figure S4). Despite the samples in the foam box being subjected to handling, vibration, and environmental perturbations (Figure 4A), the RRTC hydrogel was placed in a foam-insulated container with ice packs used as passive temperature buffers to prevent overheating, and the recorded temperature was approximately 10–30 °C (Figure S5). After transportation, the RRTC group maintained favorable cellular viability, matching the performance of the static simulation (Figure 4B,C). The use of ice packs was not intended to maintain a conventional 2–8 °C cold-chain condition or to trigger the 4 °C gel-to-sol transition during transport. These results suggest that RRTC may support short-term cryogen-free cell transportation under passively temperature-buffered conditions. However, mechanical stress during courier transport was not quantitatively monitored, which remains a limitation of this study.

### 2.4. Cell Recovery and Re-Culture of Recovered Cells

**Figure 5:**
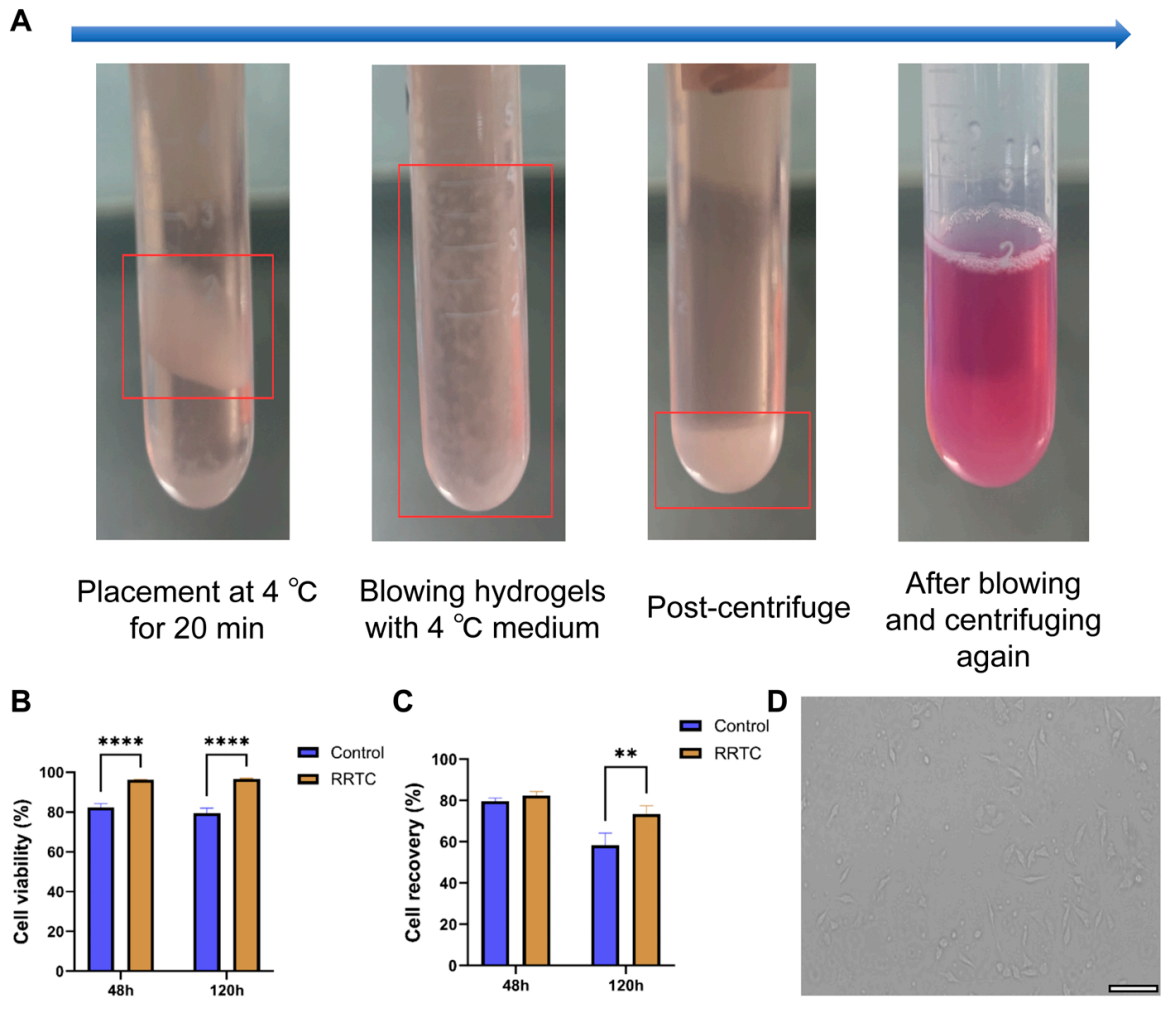
Characterization of cell recovery and re-culture after transportation. (**A**) Images showing the cell recovery status of the RRTC group. (**B**,**C**) Comparative analysis of post-transport cellular viability (**B**) and cell recovery yields (**C**) between the RRTC group and the 2D liquid-filled control group after 48 h and 120 h of transportation (*n* = 3). ** *p* < 0.01, **** *p* < 0.0001. (**D**) Bright-field image of the re-adhesion of post-transport cells from the RRTC group. Re-adhesion was used only as an indicator of basic re-culture ability and does not demonstrate long-term phenotypic preservation. Scale bar = 200 μm.

A critical bottleneck in conventional 3D cell carriers is the need for harsh enzymatic digestion to harvest cells, which strips cell-surface receptors [29,30]. The thermoresposive behavior of the RRTC system enabled an enzyme-free cell retrieval process. Exposing the RRTC constructs to a pre-cooled medium (4 °C) for 20 min induced gel-to-sol transition. The 4 °C step was not required during transport and was used only after transportation for recovery. Subsequent gentle pipetting and standard centrifugation (1000 rpm, 5 min) effectively isolated the cells (Figure 5A; Video S3).

Quantitative analyses confirmed that cells recovered from the RRTC matrix after 48 h and 120 h of transit maintained high viability (95.24 ± 1.12%) compared with the 2D liquid controls (Figure 5B). As shown in Figure 5C, after a short-term transportation of 48 h, the cell recovery yield was 79.67 ± 1.53% for the conventional 2D liquid-filled control group and 82.33 ± 2.08% for the RRTC hydrogel group, showing no statistical difference between the two environments. However, when the transit duration was extended to 120 h, the cell recovery yield of the 2D liquid-filled control group significantly reduced to 58.33 ± 5.86%, primarily due to severe cell sedimentation, multi-layer aggregation, and subsequent anoikis-induced lysis within the liquid phase. In contrast, the cells encapsulated within the RRTC hydrogel maintained a significantly higher recovery yield of 73.33 ± 4.16% (*p* < 0.01). Recovery was normalized to the initial cell number of each group. Further re-culture experiments showed that the recovered cells in the RRTC group reattached after reseeding, indicating basic post-transport re-culture ability (Figure 5D). However, re-adhesion alone does not demonstrate long-term phenotypic preservation or functional integrity. In addition, the recovery yield indicates that cell release from the hydrogel is incomplete and requires further optimization, particularly after longer transport periods.

## 3. Conclusions

The RRTC hydrogel serves as a potential matrix for short-term cryogen-free cell logistics under the tested conditions. Driven by its temperature-dependent phase transitions, the RRTC hydrogel undergoes a rapid sol-to-gel conversion at 37 °C, forming a 3D physical matrix that provides structural support and spatial confinement, shielding cells from transit-induced mechanical stress. Post-transport treatment with a pre-cooled medium at 4 °C triggers the intrinsic phase reversibility of the matrix, enabling an enzyme-free, non-destructive cell retrieval process that preserves basic post-transport cell viability and re-culture capacity. However, this study remains a preliminary proof-of-concept investigation restricted to a single murine fibroblast cell line (L929) to establish initial methodological feasibility. Systematic evaluations involving diverse cell types, extended transport durations, standardized multi-axis vibration profiles, severe temperature fluctuations, and multi-batch quality-control assessments, alongside further investigation of the system’s operational dependence on terminal refrigeration for cell recovery, are mandatory to fully transition this platform toward broader biomedical and clinical applications.

## 4. Materials and Methods

### 4.1. Materials

The RRTC hydrogel was prepared according to PCT/CN2021/125224 (Chinese patent: CN112354013B). Briefly, simulated body fluid (SBF, Chengdu Junxing Biotechnology Co., Ltd., Chengdu, China) was added to the collagen solution (Chengdu Junxing Biotechnology Co., Ltd.) to obtain the RRTC hydrogel, with a final collagen concentration of 1.4 wt%. Bovine type I collagen solution (3.5% gel) was purchased from Hebei Junxing Biotechnology Co., Ltd. (Handan, China). The final collagen concentration of 1.4 wt% was selected as the working concentration according to the material protocol and handling requirements for cell encapsulation and transportation; concentration-dependent effects were not systematically evaluated in this study.

### 4.2. Phase Transition Test of Hydrogel

To preliminarily evaluate the thermoresponsive phase-transition behavior of the RRTC hydrogel, RRTC solution and 1.4% type I collagen solution were alternately placed in a 4 °C refrigerator and a 37 °C incubator for repeated temperature-cycling treatment. The physical state of each sample at different temperatures was observed, and the transitions from the sol to the gel state and back to the sol state were recorded.

### 4.3. Thermoresponsive Rheological Characterization of Hydrogel

The rheological properties of the RRTC hydrogel and 1.4% type I collagen solution were evaluated using a temperature-controlled stress rheometer (MCR302, Anton Paar, Styria, AUSTRIA). Samples were evenly spread onto the testing platform and measured using a PP25 parallel-plate geometry. The oscillation frequency was set at 1 Hz, and the strain was fixed at 5% based on preliminary testing conditions and previous hydrogel rheology protocols. Because a complete strain-sweep test was not included, direct confirmation of the linear viscoelastic region is acknowledged as a limitation. Rheological measurements were performed using two independent temperature-cycling protocols. For the rapid temperature-cycling test, both heating and cooling rates were set at 10 °C/min to approximate the rapid temperature changes during practical gelation and recovery. For the slow temperature-cycling test, both heating and cooling rates were set at 1 °C/min to evaluate the thermoresponsive behavior under more gradual temperature variation. Each sample underwent three consecutive heating–cooling cycles to assess its thermoresponsive behavior and cyclic stability [31,32].

### 4.4. SEM Observation of Hydrogel Morphology

Scanning electron microscopy (SEM, Nova Nano450, FEI, Hillsboro, OR, USA) was used to observe the microstructure of the RRTC hydrogel after cell encapsulation and to evaluate cell loading and distribution within the hydrogel. The RRTC/cell mixture was injected into a predesigned mold and incubated at 37 °C for 10 min to allow for stable gel formation. The freshly prepared hydrogel was then frozen at −80 °C and immediately fractured to expose its cross-section. After freeze-drying for 72 h, the samples were sputter-coated with gold and examined under SEM for microstructural imaging.

### 4.5. Preparation of Samples Before Cell Transportation

The NCTC clone 929 (L929) was cultured in Dulbecco’s Modified Eagle Medium (DMEM) supplemented with 10% (*v*/*v*) fetal bovine serum and 1% (*w*/*w*) penicillin–streptomycin at 37 °C in a 5% CO_2_ environment. L929 was obtained from the cell bank of the Chinese Academy of Sciences (Shanghai, China). Before simulated transportation, cells from the same passage and batch were subjected to live/dead staining to evaluate their baseline viability. For the experimental group, cell pellets (1 × 10^7^ cells per sample) were mixed thoroughly with 0.5 mL of RRTC solution in a 15 mL centrifuge tube, followed by incubation at 37 °C for 5 min to induce gelation. After gel formation, 13 mL of culture medium prewarmed to 37 °C was added to fully immerse the cell-laden hydrogel structure.

For control group 1, a conventional two-dimensional liquid-filled transport mode was used, in which cells were cultured in T25 flasks containing an appropriate volume of culture medium and transported at room temperature. For control group 2, a conventional cryopreservation-based transport mode was used, in which cells were mixed with 1 mL of cryopreservation medium, stored at −80 °C, and transported in an insulated container filled with 5 kg of dry ice. All containers were sealed with Parafilm before simulated transportation. The experimental group and control group 1 were maintained at room temperature, whereas control group 2 was kept at low temperature. A temperature data logger continuously recorded the temperature changes in the experimental group and control group 1 during transport. All cells used in the experiments were derived from the same passage. Four parallel samples were prepared for each group. The initial number of transported cells was 1 × 10^7^ cells per sample in the experimental group and control group 2, and 2 × 10^6^ cells per sample in control group 1. The higher initial cell number in the RRTC group and cryopreservation-based control group was used to simulate high-density cell transportation, whereas a lower initial cell number was used in the 2D liquid-filled control group because excessive cell density in T25 flasks may cause cell sedimentation, aggregation, and nutrient/oxygen limitations during transportation. Cell recovery yield was normalized to the initial cell number of each group. A 3D non-thermoresponsive hydrogel control was not included in the cell transportation experiments because such a matrix would remain in a gelled state during post-transport cell recovery, thereby necessitating harsh enzymatic digestion or chemical disruption for cell release. These additional treatments could introduce confounding effects and reduce comparability with the enzyme-free recovery process of RRTC.

### 4.6. Static Simulation of Cell Transportation at Room Temperature

L929 cells were used to establish a static room-temperature transportation model. Samples from different groups were placed at room temperature without agitation to simulate conventional non-cryogenic transportation conditions. During transportation, temperature changes were continuously recorded with a temperature logger, and the cellular status of each group was monitored throughout.

### 4.7. Actual Cell Transportation Experiment

To further evaluate the feasibility of this system under practical transportation conditions, an actual transportation experiment was conducted using SF Express standard delivery for a round trip between Chengdu and Chongqing, with a total transportation time of approximately 50 h. Temperature changes during transportation were continuously recorded using a temperature logger. Samples from the experimental group and control group 1 were placed together in a foam-insulated container containing ice packs. The ice packs were included to prevent excessive ambient temperatures from adversely affecting cell viability. They were used only as passive temperature buffers and were not intended to maintain a conventional 2–8 °C cold-chain condition or to trigger the 4 °C gel-to-sol transition during transportation. After transportation, cell status and subsequent viability were evaluated.

### 4.8. Live/Dead Staining of Cells

Cell viability was evaluated using fluorescein diacetate (FDA)/propidium iodide (PI) live/dead staining for the RRTC/cell constructs in the experimental group and the cells in control group 1. An appropriate amount of the RRTC/cell construct was placed into a 96-well plate and washed twice with phosphate-buffered saline (PBS). Subsequently, 100 μL of FDA/PI staining solution (20 μg/mL) was added, and the samples were stained for approximately 3 min at room temperature in the dark. After removal of the staining solution, PBS was added to maintain sample moisture, and the samples were observed and imaged using an inverted fluorescence microscope (Olympus, IX73, Tokyo, Japan). Cells in control group 1 were collected by trypsinization and centrifugation, then subjected to the same staining procedure. Fluorescence images were analyzed using ImageJ (1.5t, National Institutes of Health, Bethesda, MD, USA), and live/dead cells were counted from randomly selected fields. Cell viability was calculated using the following equation:

(1) $$\mathrm{Cell}\;\mathrm{viability}=\frac{\mathrm{Number}\;\mathrm{of}\;\mathrm{viable}\;\mathrm{cells}}{\mathrm{Total}\;\mathrm{number}\;\mathrm{of}\;\mathrm{cells}}\times 100$$

### 4.9. Cell Recovery and Counting

Cell recovery from the RRTC hydrogel was performed according to the manufacturer’s instructions by taking advantage of its thermoresponsive property at 4 °C. Briefly, precooled culture medium (4 °C) was added to the cell-laden RRTC hydrogel, followed by repeated pipetting and centrifugation (1000 rpm, 5 min) to promote hydrogel dissociation and achieve separation of the material from the cells. The recovered cells were then collected and counted. The 4 °C medium was used only during the post-transport recovery step to induce gel-to-sol transition; therefore, refrigeration or pre-cooled medium is required for cell recovery, although the transportation process itself does not rely on dry ice, liquid nitrogen, or active cryogenic refrigeration. For control group 1, cells were harvested using the standard procedure for adherent cells: the culture medium was removed, cells were detached with trypsin, and cell pellets were obtained by centrifugation. For control group 2, cells were recovered using a conventional thawing procedure: cryovials were rapidly thawed at 37 °C, followed by centrifugation at 1000 rpm for 5 min to obtain cell pellets after removal of the supernatant. The cell recovery rate was calculated as follows:

(2) $$\mathrm{Cell}\;\mathrm{recovery}\;\mathrm{rate}=\frac{\mathrm{Number}\;\mathrm{of}\;\mathrm{cells}\;\mathrm{after}\;\mathrm{transportation}}{\mathrm{Number}\;\mathrm{of}\;\mathrm{initially}\;\mathrm{seeded}\;\mathrm{cells}}\times 100$$

### 4.10. Re-Adhesion Assay of L929 Cells After Transportation

To preliminarily assess the re-culture ability of recovered cells after transportation, the recovered L929 cells from the experimental group were seeded into 96-well plates and cultured for an additional 24 h. Cell adhesion morphology was then observed and recorded.

### 4.11. Statistical Analysis

Variables with a normal distribution are presented as the mean ± the standard deviation (S.D.). *p* values less than 0.05 were considered statistically significant (* *p* < 0.05, ** *p* < 0.01, *** *p* < 0.001). All statistical analyses were performed using GraphPad Prism 10.1.2 software. For Control Group 2, some endpoints after 120 h were interpreted qualitatively because dry ice had completely sublimated and the samples no longer represented a stable cryogenic transportation condition.

## Data Availability

The original contributions presented in this study are included in the article/Supplementary Materials. Further inquiries can be directed to the corresponding authors.

## Supplementary Materials

The following supporting information can be downloaded at https://www.mdpi.com/article/10.3390/gels12060488/s1, Figure S1. Bright field image of cells in Control Group 1 (2D liquid-filled transportation). Figure S2. Photographs of Control Group 2 (dry ice transportation). Figure S3. Live/dead fluorescence images of cells in Control Group 2 (dry ice transportation). Figure S4. Courier shipping receipt. Figure S5. Temperature variation during transportation. Videos S1–S3: Injectability of RRTC hydrogel (S1), structural integrity after mixing with cells (S2), and cell retrieval process (S3).
