# Supplementary material for: Reversible Sol–Gel Transition in Thermoresponsive Collagen Hydrogels for Cryogen-Free Cell Logistics
Source: Gels. 2026 Jun 2;12(6):488. doi: 10.3390/gels12060488 (PMC13297883; doi:10.3390/gels12060488)
Supplement: Supplementary file 1 [file gels-12-00488-s001.zip › Supplementary Information.pdf]

Reversible Sol–Gel Transition in Thermoresponsive Collagen Hydrogels for Cryogen-Free Cell Logistics

Junjie Wang [1], Yi Ju [1], Yang Lei [2], Jieyu Zhang [1,*] and Yunbing Wang [1,*]

[1] National Engineering Research Center for Biomaterials, College of Biomedical Engineering,
Sichuan University, No. 29 Wangjiang Road, Chengdu 610065, China;
w328313530@163.com (J.W.); 2025323100013@stu.scu.edu.cn (Y.J.)

[2] Institute of Biomedical Innovation, School of Basic Medical Sciences, Jiangxi Medical College,
Nanchang University, No. 1299, Xuefu Road, Honggutan District, Nanchang 330031, China;
leiyang@ncu.edu.cn

* Correspondence: jieyu@scu.edu.cn (J.Z.); yunbing.wang@scu.edu.cn (Y.W.)

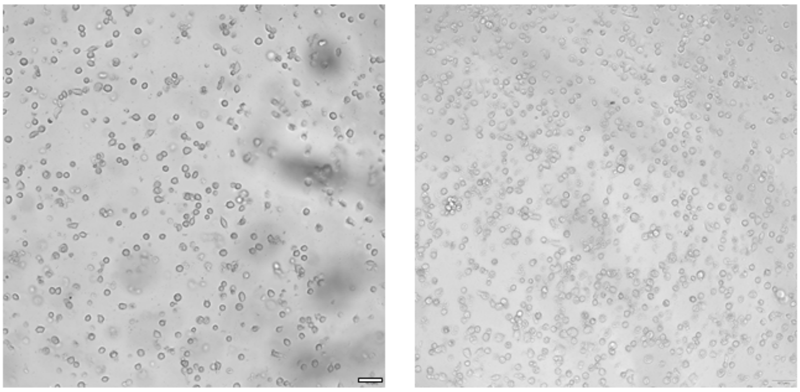

Figure S1. Bright field image of cells in the control 1 group.

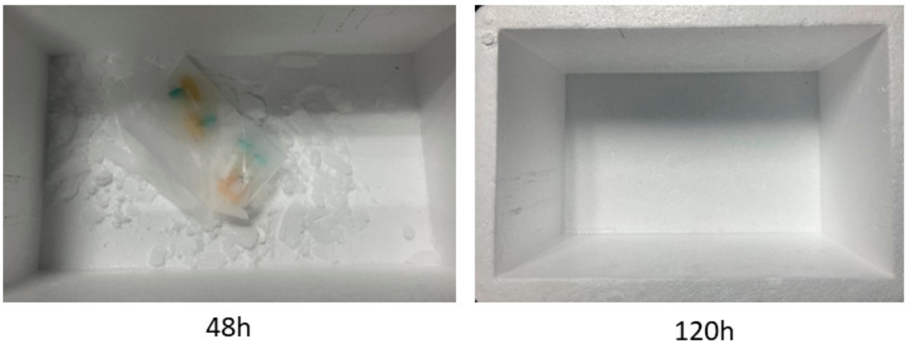

Figure S2. Schematic illustration of the dry ice transport control 2 group.

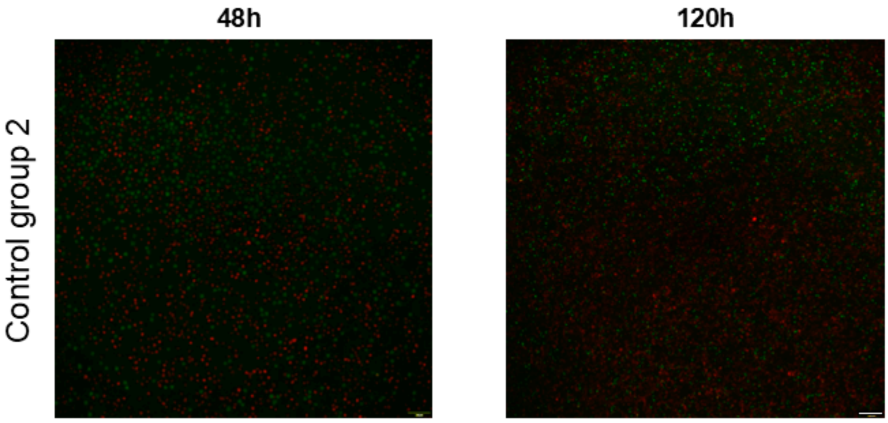

Figure S3. Live/dead fluorescence images of cells in Control Group 2.

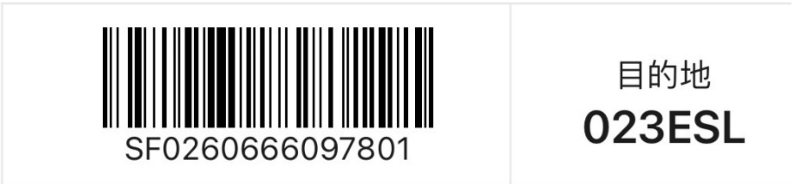

Figure S4. Courier shipping receipt. The specific Chinese characters depicted in the right section of the image translate directly to “destination”.

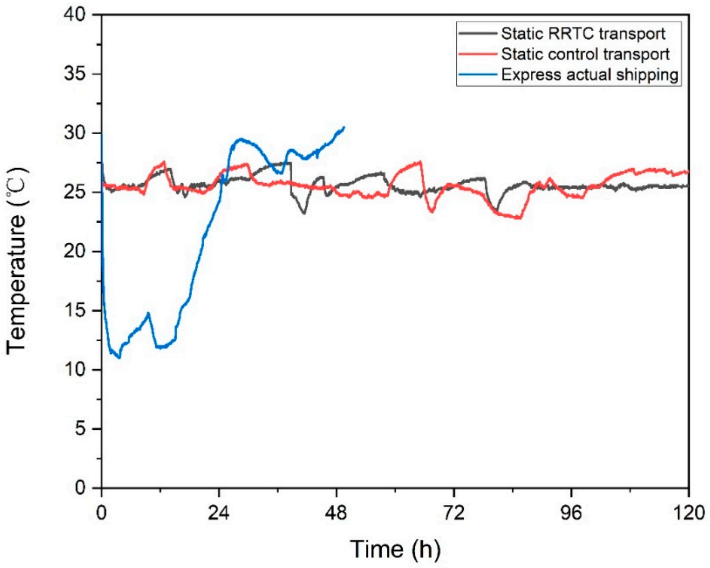

Figure S5. Temperature variation during transportation.
